# IgG *N-*glycosylation from Patients with Pemphigus Treated with Rituximab

**DOI:** 10.3390/biomedicines10081774

**Published:** 2022-07-22

**Authors:** Guillaume Font, Marie-Laure Walet-Balieu, Marie Petit, Carole Burel, Maud Maho-Vaillant, Vivien Hébert, Philippe Chan, Manuel Fréret, Olivier Boyer, Pascal Joly, Sébastien Calbo, Muriel Bardor, Marie-Laure Golinski

**Affiliations:** 1 Université de Rouen Normandie, Inserm U1234, CHU Rouen, Department of Dermatology, F-76000 Rouen, France; guillaume.font@chu-rouen.fr (G.F.); maud.maho@univ-rouen.fr (M.M.-V.); vivien.hebert@chu-rouen.fr (V.H.); pascal.joly@chu-rouen.fr (P.J.); 2 Université de Rouen Normandie, Laboratoire Glyco-MEV UR 4358, SFR Normandie Végétal FED 4277, Innovation Chimie Carnot, F-76000 Rouen, France; marie-laure.walet-balieu@univ-rouen.fr (M.-L.W.-B.); carole.burel@univ-rouen.fr (C.B.); muriel.bardor@univ-rouen.fr (M.B.); 3 Université de Rouen Normandie, Inserm U1234, F-76000 Rouen, France; mariepetit1994@gmail.com (M.P.); sebastien.calbo@univ-rouen.fr (S.C.); 4 Université de Rouen Normandie, INSERM US 51, CNRS UAR 2026, HeRacLeS-PISSARO, Normandie Université, F-76000 Rouen, France; philippe.chan@univ-rouen.fr; 5 Université de Rouen Normandie, Inserm U1234, CHU Rouen, Department of Rhumatology, F-76000 Rouen, France; manuel.freret@inserm.fr; 6 Université de Rouen Normandie, Inserm U1234, CHU Rouen, Department of Immunology and Biotherapy, F-76000 Rouen, France; olivier.boyer@chu-rouen.fr; 7 Université de Lille, CNRS, UMR 8576-UGSF-Unité de Glycobiologie Structurale et Fonctionnelle, F-59000 Lille, France

**Keywords:** pemphigus, rituximab, IgG, glycosylation, *N*-glycome, *N*-glycans, sialic acid

## Abstract

Pemphigus is a life-threatening auto-immune blistering disease of the skin and mucous membrane that is caused by the production of auto-antibodies (auto-Abs) directed against adhesion proteins: desmoglein 1 and 3. We demonstrated in the “Ritux3” trial, the high efficacy of rituximab, an anti-CD20 recombinant monoclonal antibody, as the first-line treatment for pemphigus. However, 25% of patients relapsed during the six-month period after rituximab treatment. These early relapses were associated with a lower decrease in anti-desmoglein auto-Abs after the initial cycle of rituximab. The *N-*glycosylation of immunoglobulin-G (IgG) can affect their affinity for Fc receptors and their serum half-life. We hypothesized that the extended half-life of Abs could be related to modifications of IgG *N-*glycans. The IgG *N-*glycome from pemphigus patients and its evolution under rituximab treatment were analyzed. Pemphigus patients presented a different IgG *N-*glycome than healthy donors, with less galactosylated, sialylated *N-*glycans, as well as a lower level of *N-*glycans bearing an additional *N-*acetylglucosamine. IgG *N-*glycome from patients who achieved clinical remission was not different to the one observed at baseline. Moreover, our study did not identify the *N-*glycans profile as discriminating between relapsing and non-relapsing patients. We report that pemphigus patients present a specific IgG *N-*glycome. The changes observed in these patients could be a biomarker of autoimmunity susceptibility rather than a sign of inflammation.

## 1. Introduction

Pemphigus is a rare, life-threatening auto-immune blistering disease of the skin and mucosa that causes painful erosions and severe weight loss. This disease is induced by the production of pathogenic auto-antibodies (auto-Abs) directed against desmoglein 1 (DSG1) and desmoglein 3 (DSG3), two proteins located in desmosomes and involved in keratinocytes adhesion. The interaction between auto-Abs and their target antigen induces structural changes that lead to a loss of keratinocytes adhesion, called “acantholysis”, which results in the formation of skin and mucosal blisters. There are two main forms of pemphigus: pemphigus vulgaris (PV) characterized by preferential mucosal involvement and the presence of auto-Abs that are mainly directed against DSG3, and pemphigus foliaceus (PF) characterized by exclusive skin lesions associated with the presence of anti-DSG1 auto-Abs [1,2].

Until recently, high doses of oral corticosteroids (CS) sometimes combined with immunosuppressive drugs (azathioprine, mycophenolate mofetil) were the mainstay of treatment for pemphigus [3]. The randomized, controlled clinical trial “Ritux 3” demonstrated the interest in the first-line use of rituximab (RTX) in the treatment of pemphigus. In the “Ritux 3” clinical trial, 89% of patients treated with RTX and the short-term CS (prednisone) arm were in complete remission and off therapy after 2 years, compared to 34% of patients treated with CS (prednisone) alone (*p* < 0.001) [4].

During this clinical trial, nine patients (22%) relapsed during the 12-month period following the initial infusion of RTX. As predictors of relapse, we identified an initial Pemphigus Disease Area Index (PDAI) score ≥45 and the persistence of anti-DSG1 and anti-DSG3 auto-Abs three months after the initial cycle of RTX [5]. These results suggest that the relapses might be related to more persistent anti-DSG1 and anti-DSG3 auto-Abs secreting plasma cells or a longer immunoglobulin-G (IgG) half-life. Several mechanisms have been proposed to explain the second hypothesis, such as FcRn polymorphism [6], an IgG isotype [7] or the specific modification of the IgG *N*-glycan profile [8].

*N*-glycosylation is a post-translational modification occurring on IgG, particularly at the asparagine (Asn)-297 located in the C_H_2 domain of the Fc fragment [9]. In addition, it should be noted that 15 to 25% of IgG also bear *N*-glycans on the Fab [10]. IgG *N-*glycosylation includes high heterogeneity. For example, IgG Fc *N*-glycosylation displays 36 major isoforms composed of a *N*-glycan core constituted with seven monosaccharides: four *N*-acetylglucosamine (GlcNAc) and three mannose (Man) residues. In addition, each *N-*glycan isoform differs by the types and numbers of additional monosaccharides leading to fucosylation, galactosylation and sialylation of the *N*-glycan structures as well as the addition of a bisecting GlcNAc in some cases [11]. In contrast, the human IgG1 *N*-glycan repertoire presents less heterogeneity as 11 distinct complex biantennary Fc glycoforms represent up to 90% of its *N*-glycan profile [12]. This polymorphism defines an IgG “*N*-glycome” that impacts auto-immune diseases. Indeed, an “inflammatory profile” in Crohn’s disease has been showed to be associated with lower galactosylated IgG *N*-glycans [13]. Moreover, *N*-glycome of IgG may be associated with a more severe or relapsing course, as demonstrated by the lower galactosylated *N*-glycans in patients with rheumatoid arthritis [14], or with the low galactosylation and sialylation that have been associated with active disease or relapses in patients with granulomatosis with polyangiitis and systemic lupus erythematosus [15,16,17]. It has also been shown that the removal of *N*-glycans from the Fc domain altered the pro-inflammatory activity in auto-immune mouse models [18]. Moreover, recent studies showed that sialylation could prolong the serum half-life of therapeutic monoclonal antibodies [8,19]. Furthermore, several studies have shown that the *N-*glycosylation of therapeutic intravenous immunoglobulins (IVIg) could influence their anti-inflammatory properties. Indeed, it has been demonstrated in an auto-immune mouse model that the removal of terminal sialic acid residues from IgG resulted in a loss of the anti-inflammatory activity of IVIg [20,21,22].

A transcriptomic study conducted by our team identified that the *MGAT5* gene (encoding for the alpha-1,6-mannosyl-glycoprotein beta-1,6-*N*-acetylglucosaminyltransferase), involved in *N-*glycan biosynthesis, is deregulated in the B cells of relapsing patients compared to non-relapsing patients after treatment [23]. These preliminary results indicate that a modulation of the *N-*glycan biosynthesis pathway may be involved in IgG *N-*glycosylation, and that this may have an impact on the clinical status of patients with pemphigus after treatment.

Altogether, these findings provide a rationale for studying the *N*-glycome of serum IgG from pemphigus patients and its evolution under treatment. The use of sera from pemphigus patients who were treated with RTX and short-term CS in the “Ritux 3” trial [4] allowed us to: (i) compare the IgG *N-*glycome from pemphigus patients to that from healthy donors (HD), (ii) assess the IgG *N*-glycome modification over time after RTX treatment and (iii) identify a potential relationship between IgG *N-*glycosylation and disease activity in pemphigus.

## 2. Materials and Methods

### 2.1. Patients and Controls

Sera were selected from: 13 HD; 16 pemphigus patients (14 PV and 2 PF) included in the “Ritux 3” trial [4], including 8 patients with ongoing clinical and serological remission (i.e., patients who no longer have anti-DSG auto-Abs at the Month 6 evaluation); and 8 patients who relapsed during the first year of the initial cycle of RTX and had persistent high titers of anti-DSG auto-Abs [5]. Sera from the 8 patients with sustained clinical remission were analyzed at baseline (Day 0 before treatment) and at Month 6 and Month 12 after the initial cycle of RTX. Sera from the 8 relapsing patients were analyzed at Day 0 and at the time of relapse (before the additional infusion of RTX) with a mean time to relapse corresponding to 252.3 ± 79.2 days after the initial cycle of RTX. Since these 8 patients were retreated with RTX after they relapsed, we did not perform the analysis of their IgG *N*-glycome at Month 12.

### 2.2. IgG Purification with ÄKTA-Start

IgG purification was achieved by affinity chromatography on the ÄKTA-Start system using parameters that have been previously described in [24]. In brief, the column of protein G HiTrap (GE Healthcare, Chicago, IL, USA) was balanced with 10 mL of phosphate buffer saline (PBS) 1 X, at pH 7.4. The pre-diluted sera (1/5) with PBS were added to the 1 mL protein G column, which was used at the flow rate of 1 mL/min and then, washed with 10 mL of PBS. IgG were eluted using 10 mL of glycine buffer 0.1 M, pH 2.7, followed by neutralization with 1 M Tris pH 9. Analysis was performed using UNICORN 7.0 software (Cytiva) to collect IgG-containing fractions. Purified IgG were quantified using a BCA protein assay kit (Pierce^TM^, Rockford, IL, USA) according to the manufacturer instructions.

### 2.3. N-glycome Analysis of IgG in Patients

*N*-glycan analysis was performed as previously reported in detail in [24]. In brief, purified, denatured IgG were de-glycosylated using peptide *N*-glycosidase (PNGase F from *Elizabethkingia miricola*, Sigma-Aldrich, St. Louis, MO, USA). De-glycosylated IgG were precipitated with 4 V of cold ethanol at −20 °C. The supernatant containing the released *N*-glycans was retrieved and evaporated under air flow. The *N*-glycans were permethylated, purified using the C18 column and finally, dried down prior to analysis by matrix-assisted laser desorption/ionization–time-of-flight mass spectrometry (MALDI-TOF MS; Ultraflextreme, Bruker Daltonics, Bremen, Germany). Then, samples were prepared as a mixture with dihydroxybenzoic acid (DHB) used as the matrix. This matrix was freshly dissolved at 20 mg/mL in an 80% methanol solution. Permethylated *N*-glycans were solubilized in an acetonitrile/0.1% trifluoroacetic acid 70/30 *v/v*. The samples and matrix were spotted in a ratio of 1/1 *v/v*. Mass spectra were acquired with an accumulation of a minimum of 10,000 shots in reflectron positive mode using a mass range of *m/z* 900–4500. The instrument was calibrated using a peptide calibration kit (ProteoMassTM, Sigma-Aldrich, St. Louis, MO, USA). MS/MS spectra were acquired for structural characterization (Figure S1). For these analyses, argon was used as collision gas (4 bars) in the LIFT cell, and 10,000 fragment spectra were accumulated with a random walk movement. Spectra were recorded with FlexControl 3.4 software and analyzed with FlexAnalysis 3.4 software (Bruker Daltonics, Bremen, Germany). Based on the *m/z* ratio, Glycoworkbench 2.1 and reports from the literature on IgG *N*-glycosylation, each ion was assigned to a *N*-glycan structure that was drawn according to the recently updated international nomenclature [25]. The threshold used for the peak peaking was adapted for each MALDI-TOF mass spectra according to the signal-to-noise ratio. The chosen value for each spectrum is indicated in the Tables S1–S3. For relative quantification analysis, we reported each *N*-glycan subtype intensity to the sum of *N*-glycome intensity to obtain *N*-glycan relative percentages.

### 2.4. Keratinocyte Dissociation Assay

A keratinocyte dissociation assay is currently the main tool for the analysis of antibody-induced acantholysis in PV in vitro [26], and it was performed as previously described in [24]. In brief, HaCaT cells were cultivated in 24-well plates with DMEM containing GlutaMAX (Gibco, Grand Island, NY, USA) and CaCl_2_ (1 mM) in a controlled atmosphere (CO_2_ 5%) at 37 °C. Twenty-four hours after reaching the confluence, positive control (AK23: 10 µg/mL), purified IgG from HD (62.5 µg/mL) or from PV patients (62.5 µg/mL) were added and incubated for 24 h. The HaCaT cells were then treated with a dispase solution (2.4 U/mL; Sigma-Aldrich, St. Louis, MO, USA) at 37 °C until the monolayers were separated from the plate. The monolayers were stained with crystal violet (Sigma-Aldrich, St. Louis, MO, USA) and mechanical stressed by pipetting 7 times with a 1 mL pipette. After the fixation of cell fragments, pictures were taken, and the number of fragments were counted manually. Each experiment was performed in triplicate.

### 2.5. Statistical Analysis

All statistical analyses were performed using GraphPad Prism (GraphPad Software, La Jolla, CA, USA). Comparisons of IgG *N*-glycomes between HD and pemphigus patients were performed before and after RTX treatment and short-term CS, using an unpaired *t*-test. A Wilcoxon paired test was used to compare the evolution of IgG *N*-glycomes in all pemphigus patients (*n* = 16) at baseline to Month 6 after RTX and short-term CS, and between baseline and Month 12 in non-relapsing patients (*n* = 8). *N-*glycosylation profiles in relapsing and non-relapsing pemphigus patients after RTX and short-term CS treatment were compared using the non-parametric Mann–Whitney test. Correlations between the PDAI score and *N*-glycan proportions were assessed using Pearson’s rank correlation coefficient. Differences were considered significant when *p* cor. < 0.006 with Bonferroni adjustment.

## 3. Results

### 3.1. Population of Patients

Sixteen patients with pemphigus were included in the present study. Characteristics of the patients such as age, gender, type of pemphigus as well as the clinical and immunological specificities are reported in Table 1. All of them presented with circulating anti-DSG1 and/or anti-DSG3 auto-Abs at baseline (before RTX and short-term CS treatment), with mean ELISA values of 372.3 ± 221.0 IU/mL and 1215.1 ± 427.6 IU/mL, respectively. The main clinical characteristics, blood B cell frequencies and serum anti-DSG1 and anti-DSG3 auto-Abs ELISA values of relapsing and non-relapsing patients are indicated in Table 1. For comparison, the sera of 13 healthy donors (HD) were analyzed in this study. However, for these sera, no information regarding age and gender was available.

### 3.2. N-glycan Profile of Pemphigus Patients at Baseline Compared to Healthy Donors

**Figure 1:**
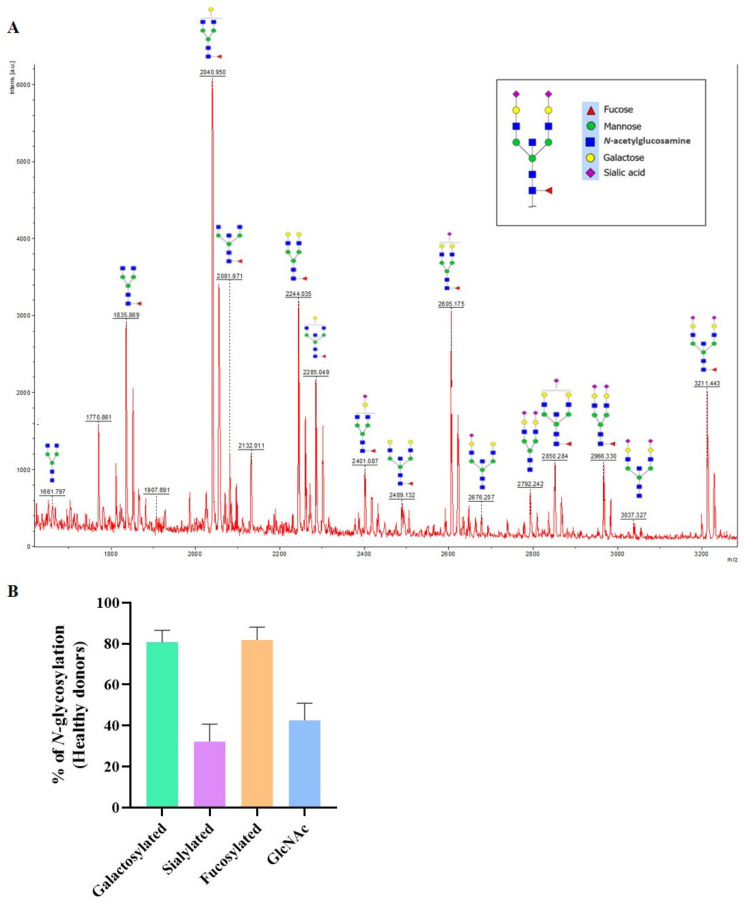
MALDI-TOF mass spectrum of total IgG *N-*glycans from healthy donors (**A**) with the proportion for each *N-*glycans subtype in the healthy donor population (*n* = 13) (**B**). Each *N-*glycan identified has been drawn according to the international nomenclature [25]. *N-*glycans were cleaved off from IgG using peptide-*N-*glycosidase F, then purified and permethylated before MALDI-TOF mass spectrometry. Mass spectra and data were obtained from FlexControl 3.3 and FlexAnalysis 3.3 software. The relationship between the corresponding ion and the *N-*glycan structure was confirmed based on MS-MS (Figure S1). The *x*-axis represents the mass-to-charge (*m/z*) ratio. The *y*-axis represents the relative percentage of the detected *N*-glycans.

The *N*-glycan profiles of IgG from the 13 HD were analyzed by MALDI-TOF mass spectrometry. A typical mass spectrum representative of the HD IgG *N*-glycome is presented (Figure 1A). The mass spectra MALDI-TOF raw data for each HD are presented in the Table S1. In agreement with the literature [27,28], the major *N*-glycan observed on IgG from HD was core-fucosylated (81.8%). Furthermore, 80.6% of IgG from HD were galactosylated, 42.6% presented with a bisecting GlcNAc, and 32.2% were sialylated (Figure 1B).

**Figure 2:**
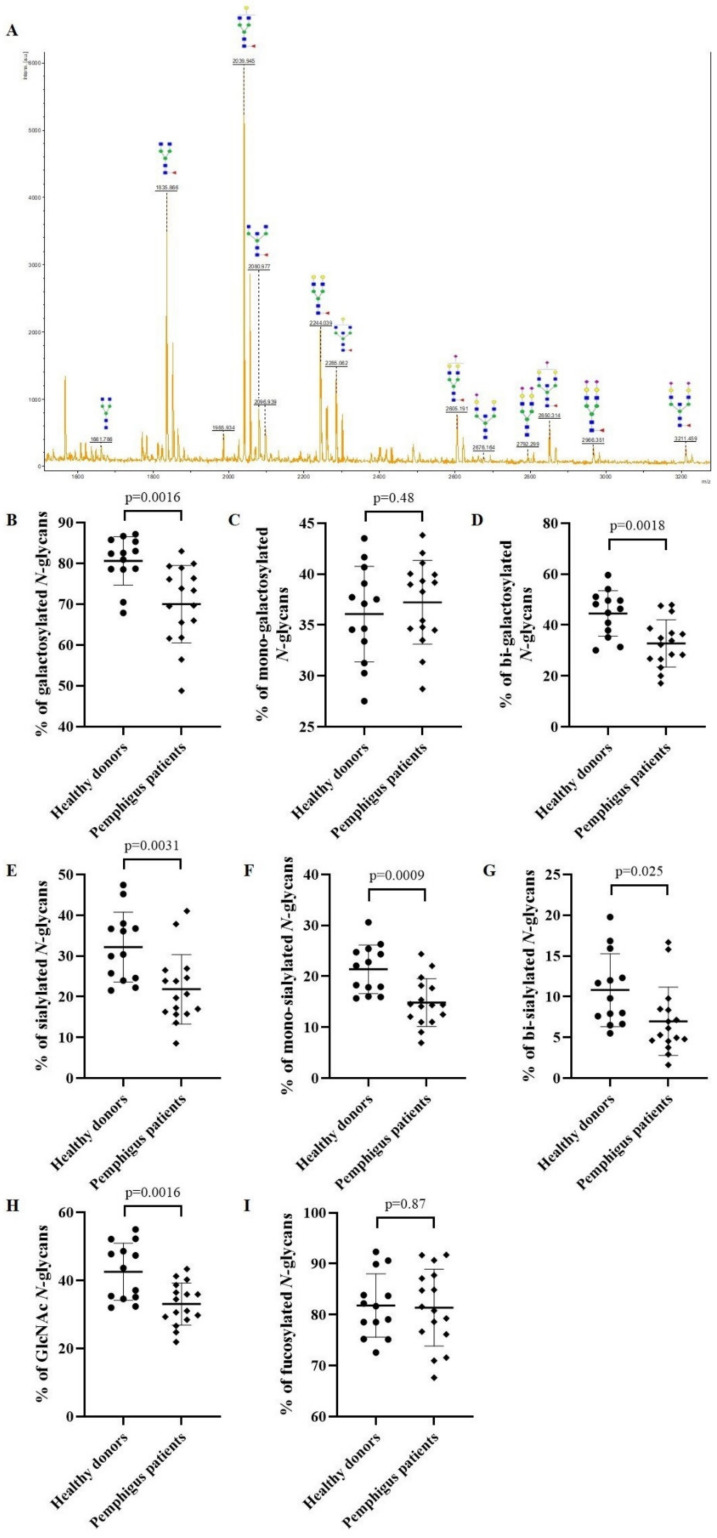
The *N-*glycan profile of pemphigus patients before rituximab treatment (at baseline) compared to healthy donors. MALDI-TOF mass spectra of total IgG *N-*glycans from pemphigus patients (**A**). The *N*-glycan structures were drawn according to the international nomenclature [25]. Blue square: *N*-acetylglucosamine; green circle: mannose; yellow circle: galactose; purple diamond: *N*-acetylneuraminic acid; red triangle: fucose. The percentage based on the relative quantification of galactosylated (**B**), mono-galactosylated (**C**), bi-galactosylated (**D**), sialylated (**E**), mono-sialylated (**F**), bi-sialylated (**G**), *N*-acetylglucosamine (GlcNAc) (**H**), fucosylated (**I**) *N*-glycans from IgG. Differences were considered significant when *p* cor. < 0.006 with Bonferroni adjustment.

Then, the *N*-glycan profiles of IgG from 16 pemphigus patients with active disease (before treatment) were analyzed by MALDI-TOF mass spectrometry. A typical mass spectrum is presented in Figure 2A. The mass spectra MALDI-TOF raw data for each pemphigus patients are presented in the Tables S2 and S3. Relative to HD, the *N*-glycans of serum IgG from pemphigus patients were less galactosylated (70.0% vs. 80.6%; *p* = 0.0016) (Figure 2B). As *N*-glycan structures can be mono- or bi-galactosylated, we determined if this galactosylation difference was related to the presence of one or two galactose residues. While the proportion of mono-galactosylated species did not differ between pemphigus patients and HD (37.2% vs. 36.1%; *p* = 0.48) (Figure 2C), the *N*-glycans of IgG of patients were significantly less bi-galactosylated compared to HD (32.8% vs. 44.5%; *p* = 0.0018) (Figure 2D). In addition, the proportion of sialylated IgG was significantly lower in pemphigus patients than in HD (21.9% vs. 32.2%; *p* = 0.0031) (Figure 2E), for mono-sialylated (14.9% vs. 21.4%; *p* = 0.0009) (Figure 2F) and with a tendency for bi-sialylated glycoforms (7.0% vs. 10.8%; *p* = 0.025) (Figure 2G).

The serum IgG of pemphigus patients also presented less *N*-glycans with a bisecting GlcNAc than IgG from HD (33.1% vs. 42.6%; *p* = 0.0016) (Figure 2H and Figure S1). Finally, fucosylation of the *N-*glycan structures did not differ significantly between IgG from patients and HD (81.4% vs. 81.8%; *p* = 0.87) (Figure 2I).

Therefore, the IgG *N*-glycome of patients at baseline was different from HD.

### 3.3. Evolution of the IgG N-glycome of Pemphigus Patients under Treatment

#### 3.3.1. Evolution of the IgG *N*-glycome under Treatment in the Whole Population of Patients

To assess the evolution of IgG *N*-glycome of pemphigus patients after RTX and short-term CS treatment, we compared the IgG *N*-glycome analyzed by MALDI-TOF mass spectrometry in sera collected at baseline (*n* = 16; 8 relapsing and 8 non-relapsing patients) to those collected after the initial cycle of RTX at Month 6 (*n* = 16; 8 relapsing and 8 non-relapsing patients) and Month 12 (*n* = 8, non-relapsing patients), respectively. Since the relapse time of the relapsing patients occurred around 6 months (252.3 ± 79.2 days) after the initial cycle of RTX, the latter were analyzed at the Month 6 time-point of the non-relapsing patients.

**Figure 3:**
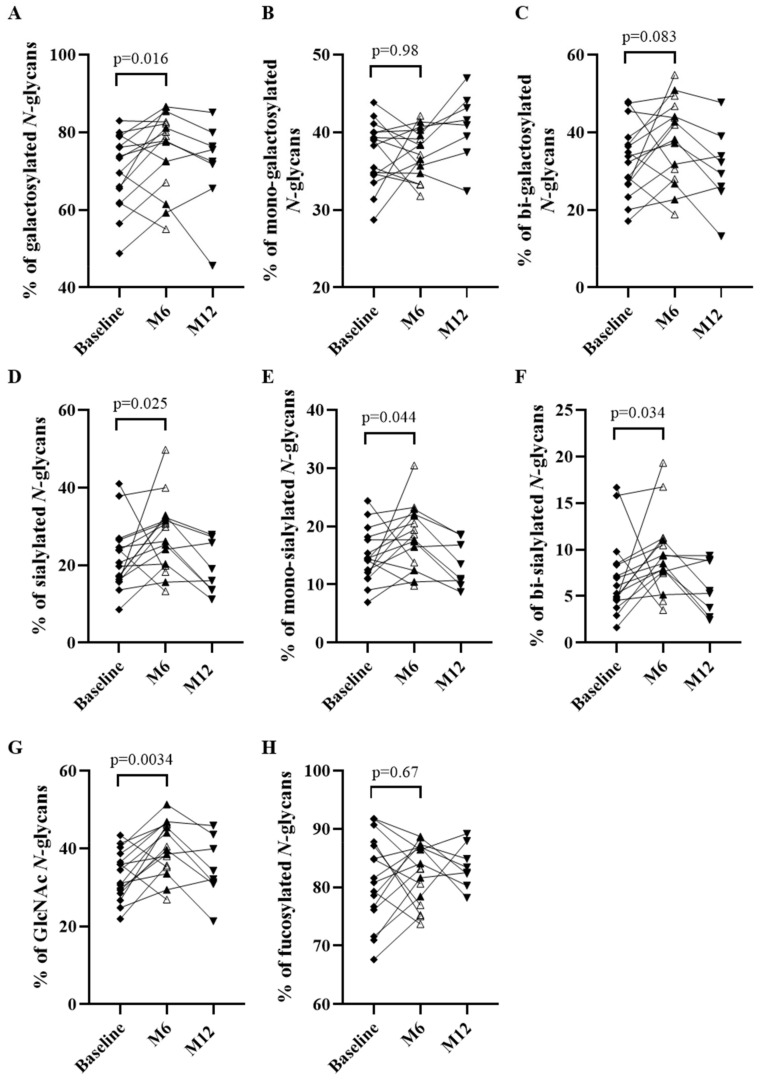
*N-*glycome evolution of pemphigus patients over time and comparison between before (baseline) and 6 months (M6) and 12 months (M12) after rituximab treatment. Comparison of percentages based on relative quantification of galactosylated (**A**), mono-galactosylated (**B**), bi-galactosylated (**C**), sialylated (**D**), mono-sialylated (**E**), bi-sialylated (**F**), *N-*acetylglucosamine (GlcNAc) (**G**), fucosylated (**H**) subgroups of *N-*glycans from IgG. Analyses were performed at baseline and M6 for all patients (*n* = 16) and at M12 only for non-relapsing patients (*n* = 8). Means ± standard deviations were compared using the Wilcoxon paired test. Differences were considered significant when *p* cor. < 0.006 with Bonferroni adjustment.

Relative to baseline, although not statistically significant, the galactosylation of the serum IgG of patients appeared to have increased at Month 6 (70.0% vs. 75.4%; *p* = 0.016) (Figure 3A) with an increasing trend for the bi-galactosylated *N*-glycan structure (32.8% vs. 38.0%; *p* = 0.083), but not for the mono-galactosylated *N*-glycans (37.2% vs. 37.4%; *p* = 0.98) (Figure 3B,C). The percentage of IgG that were sialylated seemed to have increased at Month 6 compared to baseline (21.9% vs. 27.6%; *p* = 0.025) (Figure 3D), both for mono- (14.9% vs. 18.1%; *p* = 0.044) (Figure 3E) and bi-sialylated *N*-glycans (7.0% vs. 9.5%; *p* = 0.034) (Figure 3F). In addition, we observed an increased proportion of IgG with a bisecting GlcNAc at Month 6 relative to baseline (33.1% vs. 40.2%; *p* = 0.0034) (Figure 3G). In contrast, the fucosylation of IgG did not differ between baseline and Month 6 (81.4% vs. 82.3%; *p* = 0.67) (Figure 3H).

Interestingly, we observed changes in the IgG *N*-glycomes of RTX-treated patients from baseline to Month 6, resulting in the IgG *N*-glycome of patients at Month 6 being very similar to that observed in HD IgG, in particular regarding the proportions of galactosylated (patients: 75.4%; HD: 80.6%), sialylated (patients: 27.6%; HD: 32.2%) and the presence of additional GlcNAc on IgG *N*-glycome (patients: 40.2%; HD: 42.6%) (Figure S2).

Surprisingly, the IgG *N*-glycome of non-relapsing patients was not significantly different between baseline and Month 12 (Figure 3A–H).

#### 3.3.2. Evolution of the IgG *N*-glycome Depending on Response to Treatment

**Figure 4:**
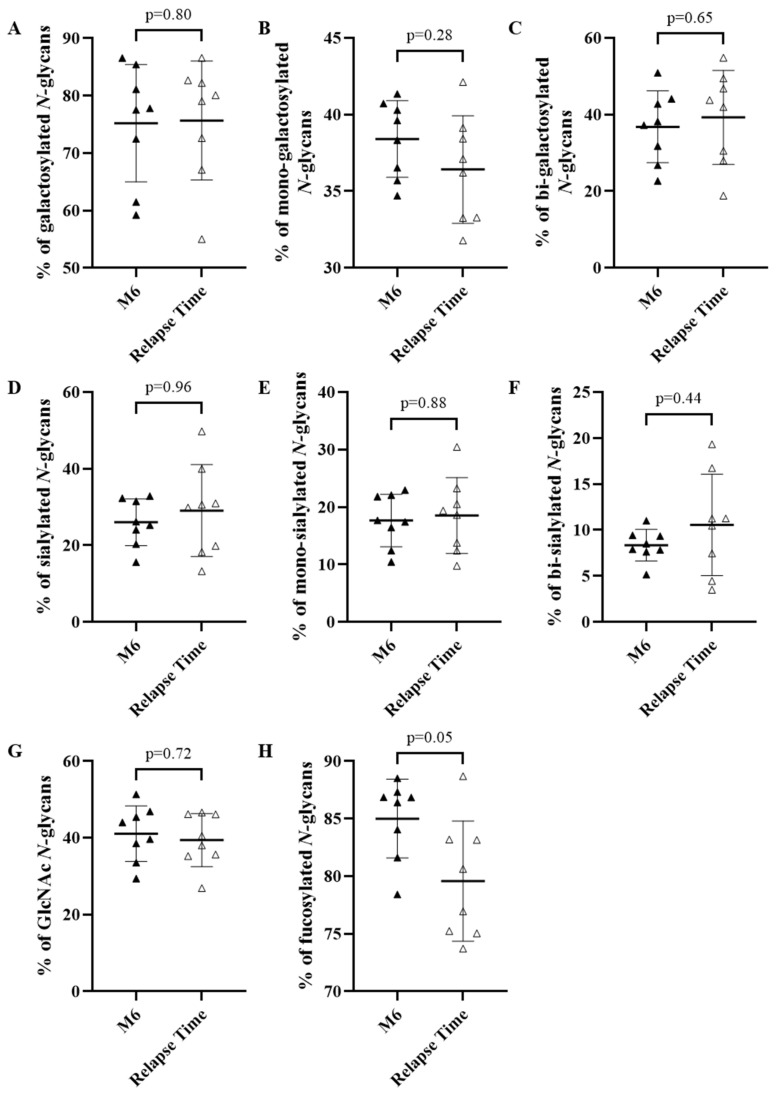
The *N-*glycome of relapsing patients during relapse occurrence compared to the *N-*glycome of non-relapsing patients at 6 months (M6). The percentage based on the relative quantification of galactosylated (**A**), mono-galactosylated (**B**), bi-galactosylated (**C**), sialylated (**D**), mono-sialylated (**E**), bi-sialylated (**F**), *N-*acetylglucosamine (GlcNAc) (**G**), fucosylated (**H**) subgroup of *N-*glycans from IgG of non-relapsing and relapsing patients for M6 and relapse occurrence, respectively. Means ± standard deviations were compared using a Mann–Whitney test. Differences were considered significant when *p* cor. < 0.006 with Bonferroni adjustment.

To assess whether a difference in the IgG *N*-glycome could be observed between sera from patients who achieved sustained complete remission after treatment and those who relapsed, we studied the IgG *N*-glycome in the serum of patients collected at Month 6 for non-relapsing patients and at the time of relapse occurrence. The proportion of galactosylated *N*-glycan structures (75.2% vs. 75.7%; *p* = 0.80) (Figure 4A–C), sialylated *N*-glycans (26.0% vs. 29.1%; *p* = 0.96) (Figure 4D–F), *N-*glycans bearing an additional GlcNAc (41.1% vs. 39.4%; *p* = 0.72) (Figure 4G) and fucosylated *N-*glycan structures (78.4% vs. 73.7%; *p* = 0.05) (Figure 4H) did not differ significantly between these two sub-populations. Similarly, no difference in the baseline *N*-glycomes was observed between patients who further relapsed and those who had a sustained clinical remission (data not shown), indicating that the *N*-glycosylation of IgG from patients was not related to whether or not patients relapsed after RTX and short-term CS treatment.

### 3.4. Pathogenic Activity of IgG of Relapsing and Non-Relapsing Pemphigus Patients

**Figure 5:**
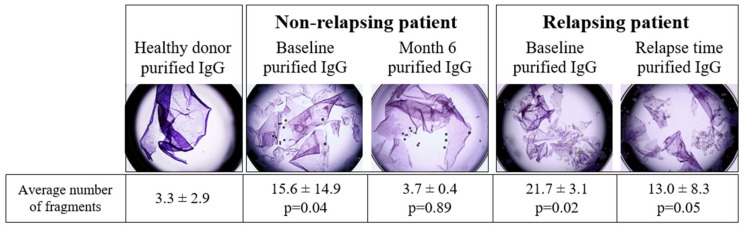
Pathogenicity evaluation of purified IgG from relapsing and non-relapsing patients at baseline and relapse time or M6, respectively. Keratinocyte dissociation assay was used to assess the pathogenicity of purified IgG from healthy donors (negative control) (*n* = 6), relapsing patients (at baseline and relapse time) (*n* = 3) and non-relapsing patients (at baseline and Month 6) (*n* = 3). In brief, HaCaT cells pre-incubated for 24 h with purified IgG were dissociated from the plate with dispase, and the monolayers were mechanically disrupted. The number of cell fragments was proportional to the IgG pathogenicity. The *p*-value was calculated in comparison to the healthy donor. A representative image of three independent experiments performed with serum from three patients is shown.

To confirm that the *N-*glycosylation profile was not related to IgG pathogenicity, we evaluated at baseline and at Month 6 the in vitro pathogenicity of purified IgG from six patients, three of whom relapsed during the evolution, while the three others remained in complete remission. No difference in the *N*-glycome of these six patients was observed both at baseline and at Month 6 (data not shown). Firstly, purified IgG collected at baseline from these six patients induced in vitro monolayer dissociation, which was significantly higher than in HD (*p* = 0.02 and *p* = 0.04, respectively) (Figure 5). Secondly, while purified IgG collected at Month 6 from the three patients in sustained complete remission had no in vitro pathogenic effect, IgG collected at the same time point from relapsing patients still induced monolayer dissociation (Figure 5). These data confirm that the IgG *N*-glycome was not correlated with the clinical status of pemphigus patients nor with the in vitro pathogenic effect of their serum.

## 4. Discussion

In the present study, we assessed the IgG *N-*glycan profile and its evolution during the course of pemphigus, an organ-specific auto-immune disease in which the pathogenic activity of auto-Abs has been demonstrated both in animal models and in keratinocyte dissociation assays. We showed that: (i) the *N*-glycome of serum IgG from patients with an active pemphigus was different from that of HD; (ii) the *N-*glycome profile of serum IgG of pemphigus patients collected at month 12 was close to the one observed at baseline; (iii) there was no major difference in the *N-*glycome of patients who relapsed after initial RTX treatment compared to those in sustained remission.

Indeed, the serum IgG *N-*glycome of patients with an active pemphigus was characterized by a decrease in the proportion of galactosylated, sialylated *N-*glycan structures as well as the ones bearing an additional GlcNAc relative to HD. Our results are in agreement with those previously reported for rheumatoid arthritis [29]. A decrease in the proportion of galactosylated and sialylated *N-*glycan structures was also observed in systemic lupus erythematosus patients relative to HD, with, nevertheless, an increase in the proportion of *N-*glycans bearing an additional GlcNAc [17]. The decrease in sialylated *N*-glycans from the IgG of pemphigus patients was directly related to the decrease in galactosylation. Many studies have reported that the lack of galactose and sialic acid residues was related to inflammation [29,30]. We did not observe any significant modification of fucosylation as previously described in other auto-immune diseases with an underlying ADCC (antibody-dependent cell-mediated cytotoxicity) mechanism [17,31,32]. Indeed, fucosylation induces a 100-fold decrease in the affinity of IgG for the FcγRIIIA, thus avoiding an excessive ADCC response [33,34,35,36]. However, such modifications of IgG fucosylation have rarely been reported in auto-immune diseases that do not involve an ADCC mechanism such as pemphigus [31].

We also observed a trend in the modification of the *N-*glycome of serum IgG from pemphigus patients after treatment with RTX and short-term CS. Indeed, analyses of the serum IgG samples collected at Month 6 showed a significant increase of *N-*glycan structures bearing a bisecting GlcNAc residue as well as a slightly increased proportion of galactosylated, sialylated *N-*glycans than at baseline in patients with an active disease. Although these findings were not statistically significant, they agreed with the increase in galactosylation and sialylation reported in the *N-*glycan structures of patients with rheumatoid arthritis after treatment with methotrexate [37]. These changes could be explained by the depletion of circulating B cells 6 months after RTX and short-term CS, whereby the IgG *N-*glycans analyzed were derived only from long-lived plasma cells. Indeed, we previously showed that the initial infusion of RTX induced a dramatic decrease in peripheral whole blood B cells at Month 6, followed by a transient re-increase from Month 9 to Month 12 and associated with a disappearance of serum IgG anti-DSG Abs [38].

However, few *N-*glycans differences were reported in patients with auto-immune thrombocytopenia treated with RTX [39]. Indeed, although IgG *N-*glycosylation in this auto-immune disease was similar to HD and did not predict treatment responses to RTX, a modification of IgG *N-*glycome was reported 2 months after RTX treatment compared to baseline.

Interestingly, the modifications of the *N-*glycome that we observed between baseline and Month 6 seemed transient, since the *N*-glycome profile of serum IgG collected at Month 12 was close to that observed at baseline. As CS treatment was stopped at Month 6 for most patients in this study, it is likely that the modifications of IgG *N*-glycome observed between baseline and Month 6 might be related to the effect of CS rather than to RTX. Accordingly, it has been described in the collagen-induced arthritis animal model that dexamethasone modified the IgG *N-*glycosylation, resulting in a higher proportion of galactosylated *N*-glycans compared to naive mice [40]. It has also been reported that diet and/or some drug intake could modify the *N-*glycome of serum IgG [41,42] or that it could be a consequence of reduced inflammation due to general immunosuppression. Indeed, it is clearly established that agalactosylated and asialylated serum IgG is associated with inflammation, whereas the presence of galactosylated and sialylated IgG correlates with an improved inflammation [20,43,44,45]. Unfortunately, in a previous study conducted by our team on only three CS-treated patients, this hypothesis could not be confirmed due to a lack of statistical power and the inter-individual variations observed [24].

Our results do not argue for a relationship between serum IgG *N*-glycome and the pathogenic activity of corresponding sera. Firstly, we did not observe any relationship between IgG *N*-glycome and the clinical severity of pemphigus assessed at baseline (data not shown). The clinical severity of pemphigus was assessed using the PDAI scoring system as previously reported [46]. Secondly, the analysis of IgG *N*-glycome in serum collected at Month 12 in patients in clinical remission was close to that observed before treatment in patients with an active disease. Thirdly, we did not evidence any major difference between the *N*-glycome of patients who relapsed after the initial treatment and those who remained in sustained clinical remission. Finally, these observations were in accordance with the demonstration of the persistent in vitro pathogenic activity of sera from relapsing patients compared to sera from remitted patients, while both sera had a quite similar *N*-glycome.

Some studies have reported an effect of the *N-*glycosylation on disease activity in patients with inflammatory bowel disease, systemic vasculitis and rheumatoid arthritis [13,14,16], which all involve an ADCC mechanism. Indeed, it is well known that *N-*glycosylation of IgG modifies the affinity between the Fc part of the IgG and the Fcγ receptors [9]. In contrast, the absence of a relationship between pemphigus severity and modifications of IgG *N-*glycosylation might be related to the fact that ADCC is not involved in the pathogenesis of pemphigus, and that *N-*glycosylation only slightly modifies the antigen/Ab affinity [9]. Furthermore, Fab *N*-glycosylation changes might impact the antigen/Ab affinity [47], but only 15 to 25% of IgG also bear *N*-glycans on the Fab [10]. Thus, the results of our study mainly reflect the overall IgG *N*-glycans.

Finally, the results of this study agree with the findings of a recently published study conducted on CS-treated patients [24]. Although this study was only conducted on three pemphigus patients, they showed that: (i) the *N-*glycan profile of pemphigus IgG was different from that of HD but was not modified by the treatment, (ii) the pathogenicity of pemphigus IgG did not seem to be related to the variability of the IgG *N-*glycome during the course of pemphigus.

The main limitation of this study is the fact that our analyses were performed on whole serum IgG as most studies in the literature [14,16], which does not necessarily imply that our results are relevant to the population of anti-DSG auto-Abs. It should be noted that such anti-DSG auto-Abs, especially the anti-DSG3, represent above 4% of the overall IgG population. Therefore, despite several attempts to purify the anti-DSG3 auto-Abs, the quantities of anti-DSG IgG obtained after purification on affinity columns were not sufficient for *N*-glycome analyses.

Moreover, it would be interesting to analyze the IgG *N-*glycome from a higher number of HD in order to improve the statistical power of this study. Some studies have shown evidence of high inter-individual variability in the IgG *N-*glycome, which could explain the absence of statistical difference observed in some of our analyses [48,49]. These studies have also demonstrated that very few modulations of the IgG *N*-glycome were observed over time, suggesting that the modulations observed in our study were likely to be related to the treatment. In addition, we had no information about the age and gender of the HD included in our study, which would be interesting data, since many studies have shown that these two parameters can influence changes in IgG *N-*glycosylation [31,50,51,52,53]. However, the pemphigus patients included in this study constituted a heterogeneous population (age and sex) with very few co-morbidities that was comparable to the population of the French blood donors.

## 5. Conclusions

Overall, despite the fact that pemphigus patients had a different *N*-glycome from HD, and a transient modification of *N*-glycome was observed under treatment, our findings do not support a clear relationship between IgG *N-*glycosylation and disease activity in pemphigus. This might be related to the fact that pemphigus does not involve an ADCC mechanism unlike many other non-organ specific auto-immune diseases, in which modifications of IgG *N-*glycosylation have been correlated with response to treatment. These results suggest that the IgG *N*-glycome changes (i.e., a decrease in the proportion of galactosylated, sialylated *N-*glycan structures as well as the ones bearing a bisecting GlcNAc) observed in pemphigus patients before treatment in comparison with HD could be a biomarker of autoimmunity susceptibility rather than a sign of inflammation.

## Figures and Tables

**Table 1:** Clinical and immunological characteristics of relapsing and non-relapsing patients at baseline and during the course of pemphigus. Auto-Ab: auto-antibody; DSG: desmoglein; IU: international units; NA: not applicable; SEM: standard error of the mean.

	Relapse (n = 16)		
Characteristic	Yes	No	*p*-Value
Number	8	8	NA
Age, mean (SEM)	47.0 (4.7)	54.8 (5.8)	0.32
Gender, n (%)			
Male	3 (37.5)	1 (12.5)	0.57
Female	5 (62.5)	7 (87.5)	
Type of pemphigus, n (%)			
Vulgaris	6 (75)	8 (100)	0.47
Foliaceus	2 (25)	0 (0)	
PDAI score, mean (SEM)	54.2 (14.0)	37.4 (29.3)	0.35
B cell frequency, mean (SEM), %			
Baseline	19.5 (4.5)	9.1 (0.5)	0.10
Month 6	0.9 (0.5)	0.1 (0.0)	0.20
Month 12	NA	4.5 (1.5)	NA
Anti-DSG1 auto-Ab level, mean (SEM), IU/mL			
Baseline	568.6 (298.0)	176.0 (70.1)	0.22
Month 6	9.0 (6.1)	1.0 (0.0)	0.21
Month 12	NA	1.0 (0.0)	NA
Anti-DSG3 auto-Ab level, mean (SEM), IU/mL			
Baseline	1615.3 (559.6)	815.0 (181.2)	0.19
Month 6	247.3 (180.4)	6.6 (1.8)	0.20
Month 12	NA	6.6 (1.9)	NA

## Data Availability

All data are available upon request.

## Supplementary Materials

The following supporting information can be downloaded at: https://www.mdpi.com/article/10.3390/biomedicines10081774/s1, Figure S1: Examples of MALDI-TOF MS-MS spectra allowing the identification of the *N*-glycan structures assigned to the different ions. (**A**) LIFT MS-MS MALDI-TOF spectra of the ion at *m/z* 1835 corresponding to the biantennary *N*-glycan containing two terminal *N*-acetylglucosamine. (**B**) LIFT MS-MS MALDI-TOF spectra of the ion at *m/z* 2676 corresponding to a complex-type *N-*glycan bearing an additional bisecting *N*-acetylglucosamine. The *N*-glycan structures were drawn according to the international nomenclature [25]. Blue Square: *N*-acetylglucosamine; green circle: mannose; yellow circle: galactose; purple diamond: *N*-acetylneuraminic acid, red triangle: fucose; Figure S2: Pemphigus patient’s *N-*glycan profile after rituximab treatment (M6) (*n* = 16) compared to healthy donor (*n* = 13). Percentage based on relative quantification of galactosylated (**A**), mono-galactosylated (**B**), bi-galactosylated (**C**), sialylated (**D**), mono-sialylated (**E**), bi-sialylated (**F**), *N-*acetylglucosamine (GlcNAc) (**G**), fucosylated (**H**) *N-*glycans from IgG. Means ± standard deviations were compared using unpaired standard *t*-test. Differences were considered significant when *p* cor. < 0.006 with Bonferroni adjustment; Table S1: Mass spectra MALDI-TOF raw data from heathy donors; Table S2: Mass spectra MALDI-TOF raw data from non-relapsing patients; Table S3: Mass spectra MALDI-TOF raw data from relapsing patients.
