# Supplementary material for: IgG N-glycosylation from Patients with Pemphigus Treated with Rituximab
Source: Biomedicines. 2022 Jul 22;10(8):1774. doi: 10.3390/biomedicines10081774 (PMC9330150; doi:10.3390/biomedicines10081774)
Supplement: Supplementary file 1 [file biomedicines-10-01774-s001.zip › biomedicines-1764865-supplementary-figure-done.pdf]

biomedicines

MDPI

Supplementary Materials

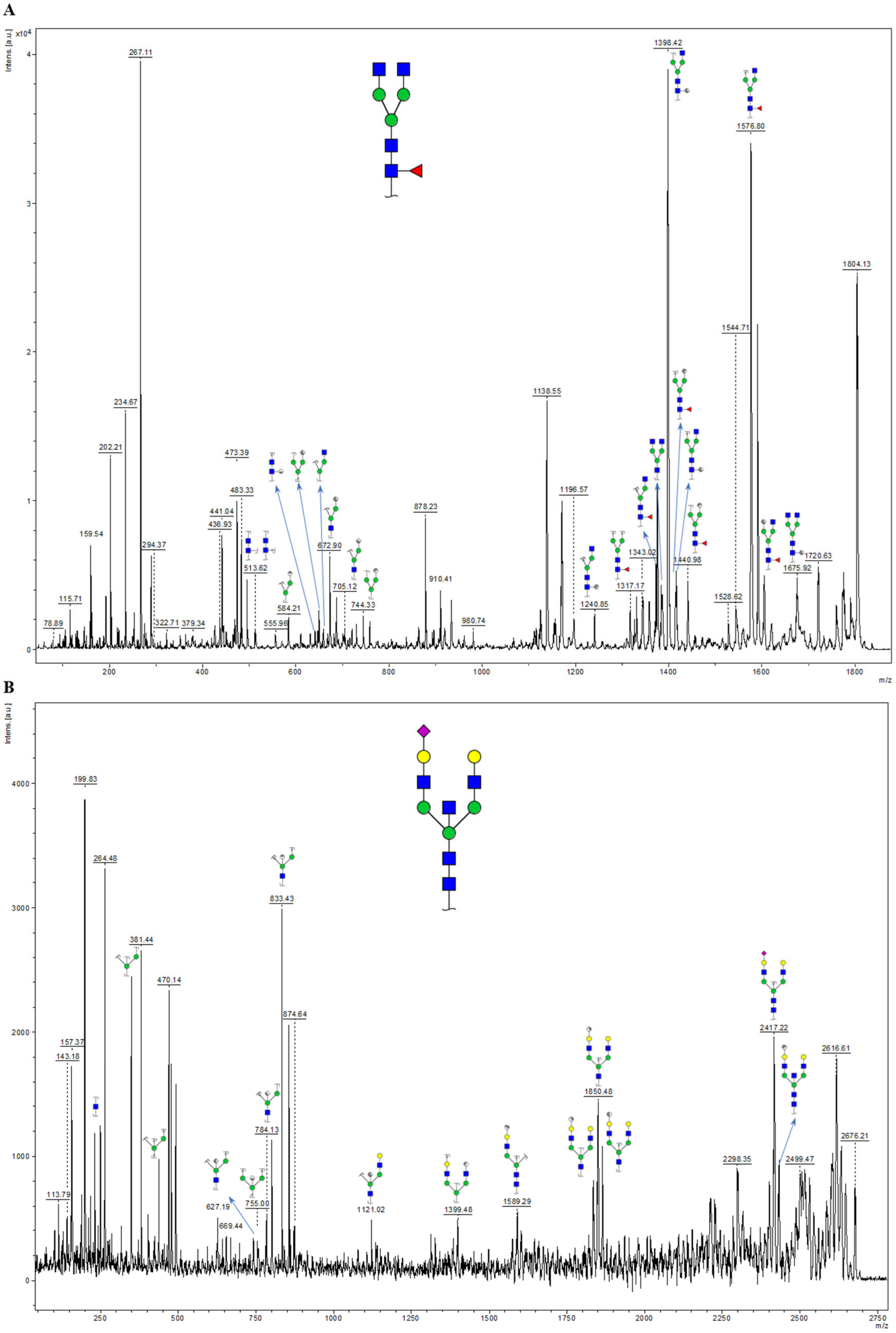

Figure S1. Examples of MALDI-TOF MS-MS spectra allowing the identification of the *N*-glycan structures assigned to the different ions. (**A**) LIFT MS-MS MALDI-TOF spectra of the ion at *m/z* 1835

corresponding to the biantennary *N*-glycan containing two terminal *N*-acetylglucosamine. (**B**) LIFT MS-MS MALDI-TOF spectra of the ion at *m*/*z* 2676 corresponding to a complex-type *N*-glycan bearing an additional bisecting *N*-acetylglucosamine. The *N*-glycan structures were drawn according to the international nomenclature [25]. Blue Square: *N*-acetylglucosamine; green circle: mannose; yellow circle: galactose; purple diamond: *N*-acetylneuraminic acid, red triangle: fucose.

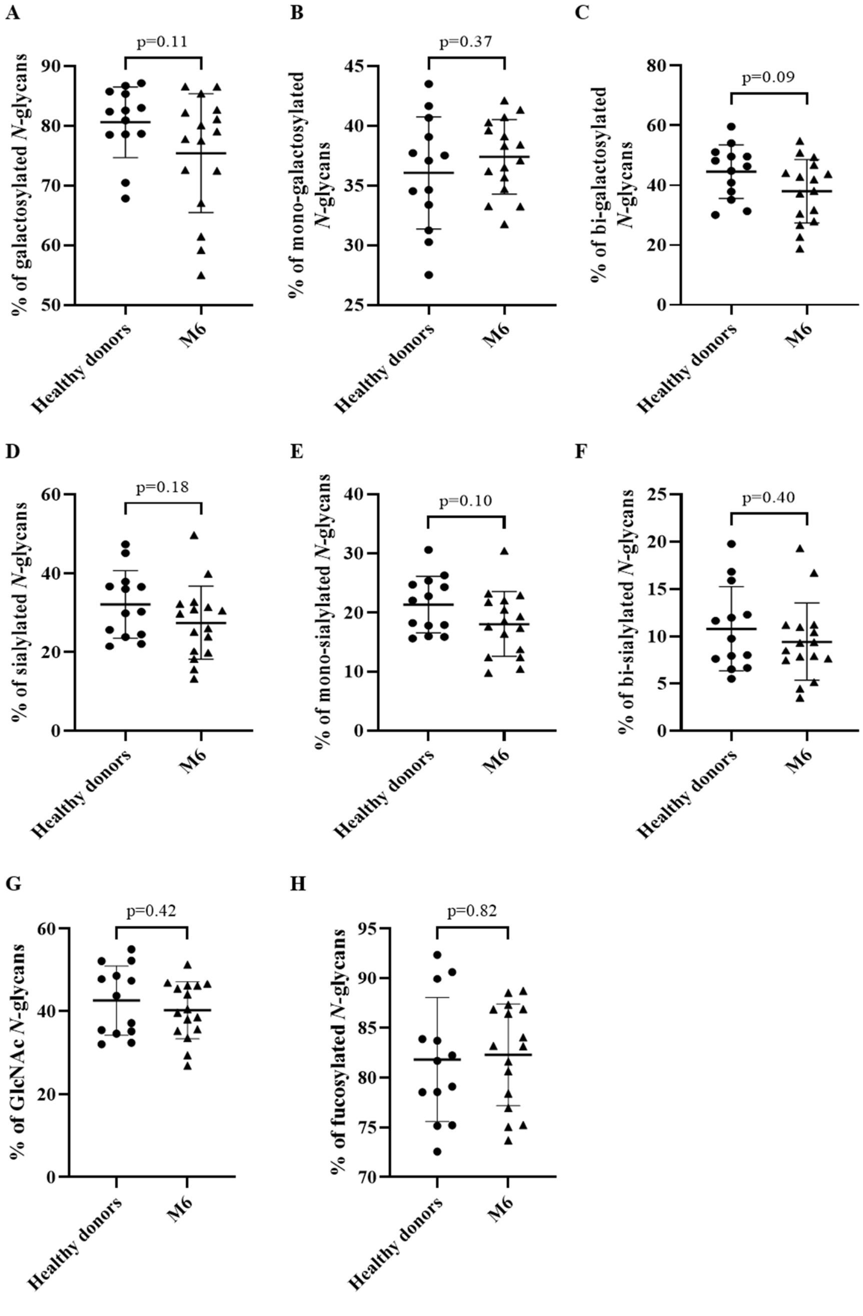

Figure S2. Pemphigus patient's *N*-glycan profile after rituximab treatment (M6) ($n = 16$) compared to healthy donor ($n = 13$). Percentage based on relative quantification of galactosylated (**A**), mono-galactosylated (**B**), bi-galactosylated (**C**), sialylated (**D**), mono-sialylated (**E**), bi-sialylated (**F**), *N*-acetylglucosamine (GlcNAc) (**G**), fucosylated (**H**) *N*-glycans from IgG. Means ± standard deviations were compared using unpaired standard t-test. Differences were considered significant when *p* cor. < 0.006 with Bonferroni adjustement.
